# Drug repositioning based on heterogeneous networks and variational graph autoencoders

**DOI:** 10.3389/fphar.2022.1056605

**Published:** 2022-12-21

**Authors:** Song Lei, Xiujuan Lei, Lian Liu

**Affiliations:** School of Computer Science, Shaanxi Normal University, Xi’an, China

**Keywords:** drug repositioning, heterogeneous network, variational graph autoencoders, graph representation learning, COVID-19

## Abstract

Predicting new therapeutic effects (drug repositioning) of existing drugs plays an important role in drug development. However, traditional wet experimental prediction methods are usually time-consuming and costly. The emergence of more and more artificial intelligence-based drug repositioning methods in the past 2 years has facilitated drug development. In this study we propose a drug repositioning method, VGAEDR, based on a heterogeneous network of multiple drug attributes and a variational graph autoencoder. First, a drug-disease heterogeneous network is established based on three drug attributes, disease semantic information, and known drug-disease associations. Second, low-dimensional feature representations for heterogeneous networks are learned through a variational graph autoencoder module and a multi-layer convolutional module. Finally, the feature representation is fed to a fully connected layer and a Softmax layer to predict new drug-disease associations. Comparative experiments with other baseline methods on three datasets demonstrate the excellent performance of VGAEDR. In the case study, we predicted the top 10 possible anti-COVID-19 drugs on the existing drug and disease data, and six of them were verified by other literatures.

## 1 Introduction

Since the outbreak of the new coronavirus pneumonia, the development of a new drug to treat the new coronavirus has become particularly important. However, traditional drug development is a high-cost, high-failure and slow process. It takes an average of 15 years from development to clinical use for an effective drug, and the economic cost is 8–1.5 billion dollars (Ashburn and Thor, 2004; Dickson and Gagnon, 2004; Adams and Brantner, 2006). Drug repurposing has the advantages of low R&D cost and short development time, so repurposing old drugs to treat common and rare diseases is becoming more and more attractive (Pushpakom et al., 2019). Drug repositioning is a strategy used to expand the applicability of older drugs (Padhy and Gupta, 2011). Those drugs that have been put into use have passed various clinical trials, and their safety and side effects have also been evaluated by relevant departments. Drug repositioning technology can shorten the development cycle to 6.5 years and the average cost to 3 million dollars (Nosengo, 2016). There is an urgent need to propose some new computational methods for drug relocation to facilitate drug development.

Traditional drug retargeting is based on biological activity, which is a wet experimental strategy that requires manual extensive analysis and testing of drugs in existing clinical compound databases. In recent years, with the continuous accumulation of high-throughput genomic and proteomic data related to drugs, many computational drug relocation methods have been generated, using some online public databases and bioinformatics tools to predict drugs, targets and interactions between diseases (Shim and Liu, 2014). At present, the existing computational drug relocation methods are divided into four categories, namely, methods based on machine learning, methods based on deep learning, methods based on network propagation, methods based on matrix decomposition and matrix completion (Luo et al., 2021).

Machine learning methods have been widely used to compute drug repositioning, usually treating drug-disease association prediction as a binary classification problem, treating drug and disease information as features. These approaches follow the principle of similarity that similar drugs are more likely to be associated with similar diseases. Gottlieb et al. (2011) proposed a computational approach, PREDICT, which constructs multiple drug-drug and disease-disease similarity measures, follows the method in Perlman et al. (2011) to construct categorical features, and then learns a logistic regression classifier to predict new links between drugs and disease. Pliakos and Vens (2020) predicted drug target interactions through tree ensemble learning and output space reconstruction. Napolitano et al. (2013) integrated information from multiple drug-related features to train a kernel-based SVM classifier. Moghadam et al. (2016) also established a support vector machine model to identify new drug-disease associations by employing nuclear fusion techniques and various features of drugs and diseases. However, the above feature-based classification methods rely heavily on the extraction of drug and disease features and the selection of negative samples. Therefore, more efficient and accurate algorithms have been developed, and matrix factorization and matrix completion techniques have been successfully applied to drug-disease association prediction. Matrix factorization methods assume that there are limited factors that determine drug, target, and disease relationships, which can be efficiently obtained by matrix factorization. Sajadi et al. (2022) predict drug-target interactions by a denoising autoencoder matrix factorization method. Dai et al. (2015) proposed a matrix factorization model to predict novel drug-disease correlations by integrating drug, gene, and disease information. Xuan et al. (2019a) developed a drug similarity-based non-negative matrix factorization model (DivePred) for predicting potential drug-disease associations. Matrix completion approaches reveal new indications by populating unknown elements in drug, target, and disease association matrices. Bagherian et al. (2021) proposed a coupled matrix-matrix completion approach to predict drug-target interactions. Luo et al. (2018) proposed a drug retargeting recommendation system (DRRS) for predicting drug-disease associations by integrating drug and disease similarity information. Compared with other methods, the above methods do not require negative samples and can flexibly integrate more prior information, but it is challenging to apply them to large-scale data due to the high complexity of matrix operations.

Deep learning is a subfield of machine learning that has been successfully applied in computer vision, speech recognition, bioinformatics, and many other fields, including prediction of drug-disease associations. Zeng et al. (2019) developed a deep learning method named deepDR. It takes full advantage of the topological information of drug similarity networks. However, deepDR does not consider disease-related information. Wang et al. (2021) proposed a deep learning model called Deep Forest multi-label classification for lncRNA disease association prediction. Yu et al. (2021) proposed LAGCN, which used graph convolutional networks to capture the feature information of drugs and diseases, and introduced an attention mechanism to combine the embeddings of different convolutional layers. Sajadi et al. (2021) proposed a deep unsupervised learning based drug-target interaction prediction method (AutoDTI++). Existing deep learning techniques mainly use the side information of drugs and diseases to build heterogeneous networks, apply deep learning techniques to heterogeneous networks to better learn the representation of drugs and diseases, and ultimately improve the prediction accuracy.

Network-based methods have become a widely used strategy in the field of computational drug relocation. The accuracy of drug repositioning is improved by capturing information similar to drug and disease characteristics in different kinds of biological networks. Luo et al. (2016) applied random walks on drug-disease dichotomous networks and drug-target-disease heterogeneous networks to predict novel drug-disease associations, respectively. Chen et al. (2012) predict drug-target interactions by random walk on heterogeneous networks. Wang et al. (2014) designed a three-layer heterogeneous network-based prediction method (TLHGBI) to infer potential links between drugs and diseases. Zeng et al. (2020) proposed a network-based arbitrary order proximity embedded deep forest method to predict drug-target interactions. Martinez et al. (2015) proposed DrugNet, a network-based prioritization method that integrates disease, drug, and target information to perform drug-disease and disease-drug prioritization simultaneously. The above methods introduce heterogeneous networks to represent the integration of different types of biological networks, and the similarities between different biological networks provides a new idea for predicting unobserved correlations between drugs and diseases. However, network-based methods focus on building heterogeneous networks while ignoring the biological knowledge of drugs and diseases. Future models that consider aspects should further improve drug-disease-association prediction.

In this article, we propose a heterogeneous network and variational graph autoencoder-based approach, VGAEDR, for predicting novel drug-disease associations. Considering the biological knowledge of drugs and diseases, we constructed three drug similarity networks and one disease similarity network based on three drug attribute information and disease semantic information, respectively, and then integrated the known drug-disease associations to construct drug- Disease Heterogeneous Networks. The VGAEDR model is divided into two parts. The first part is the Variational Graph Autoencoder (VGAE) module, which takes a heterogeneous network as input and learns and extracts its low-dimensional embedding representation. The second part is a multi-layer convolution module for further learning the embedding representation extracted by the VGAE module. Finally, the association prediction of the drug-disease pair is obtained through the fully connected layer and the softmax layer. We demonstrate through ablation experiments that three drug attribute similarities are helpful for model performance prediction. Comparative experiments with other methods on three datasets also show that our model has excellent performance. Case studies were also conducted to predict possible drugs against COVID-19.

The main contributions of this work are summarized as the following three points: 1) We propose VGAEDR, a deep learning method based on heterogeneous networks, which can effectively predict drug and disease associations. 2) VGAEDR integrates two models. Firstly, it uses a variational graph autoencoder to extract the feature representations of drugs and diseases from the drug-disease heterogeneous network, and then further learns the embedding representations of potential drugs and diseases through a convolutional neural network. 3) VGAEDR can quickly and accurately find candidate drugs against COVID-19.

## 2 Materials

### 2.1 Dataset

In order to take into account the biological association network and drug-disease-related biological knowledge at the same time, the data set in our study contains drug-disease association information and four attribute information. The four attribute information is the chemical substructure of the drug, drug target proteins and the gene annotation information for drugs, and disease semantic information of structural domains for diseases. The Comparative Toxicogenomics Database (CTD) contains many known drug-disease associations, and we screened 37,424 drug-disease associations (version 2022.7.31) from the CTD with marked therapeutic relationships, which corresponded to 6856 drugs and 2484 diseases. In order to predict the relationship between drugs and diseases in a more targeted manner, we extracted drugs that have therapeutic effects on more than 10 diseases and diseases affected by more than 10 drugs, and finally obtained 855 drugs, 727 diseases and 29,274 associations. Our study also collected two widely used benchmark datasets, the first one obtained by Zhang et al. (2018) from the CTD database, which contained 18416 known drug-disease associations between 269 drugs and 598 diseases. The second dataset is the gold standard dataset used in Liang et al. (2017), which contains 3051 known drug-disease associations between 763 drugs and 681 diseases. Our method utilizes both drug and disease similarity information, and the chemical substructures of the drug are established by obtaining the chemical fingerprints of the drug from the PubChem database (Kim et al., 2016). The domains of drug target proteins were obtained from the InterPro database (Mitchell et al., 2015). Gene annotation information for drug target proteins was obtained from the UniPort database (Renaux and UniProt, 2018). According to Wang et al. (2010), we compute the semantic similarity of diseases by constructing a directed acyclic graph (DAG) of diseases. Disease terms for constructing DAGs were obtained from the United States National Library of Medicine (ULM). The known drug-disease association is used as a positive sample set, and the unrelated drugs and diseases in the positive sample set are randomly paired to construct a negative sample set, and the number of negative samples is equal to the number of positive samples to avoid imbalance problems. Simple statistics about these two datasets are shown in Table 1.

**TABLE 1:** The statistics of three datasets.

Datasets	Drugs	Diseases	Known associations
CTD	855	727	29274
Dataset 1 Yu et al. (2021)	269	598	18416
Dataset 2 Sajadi et al. (2021)	763	681	3051

### 2.2 Construction of the heterogeneous network

In this section, we construct a drug similarity network, a disease similarity network and a drug-disease association network from the drug feature information, disease semantic information, and drug-disease association information in the above datasets, respectively. There are three kinds of drug feature information, so three drug similarity networks are constructed, which reflect the similarity of two drugs from different perspectives. Then drug-disease heterogeneous network is constructed based on the drug similarities of different classes.

#### 2.2.1 Drug-disease association network

**FIGURE 1:**
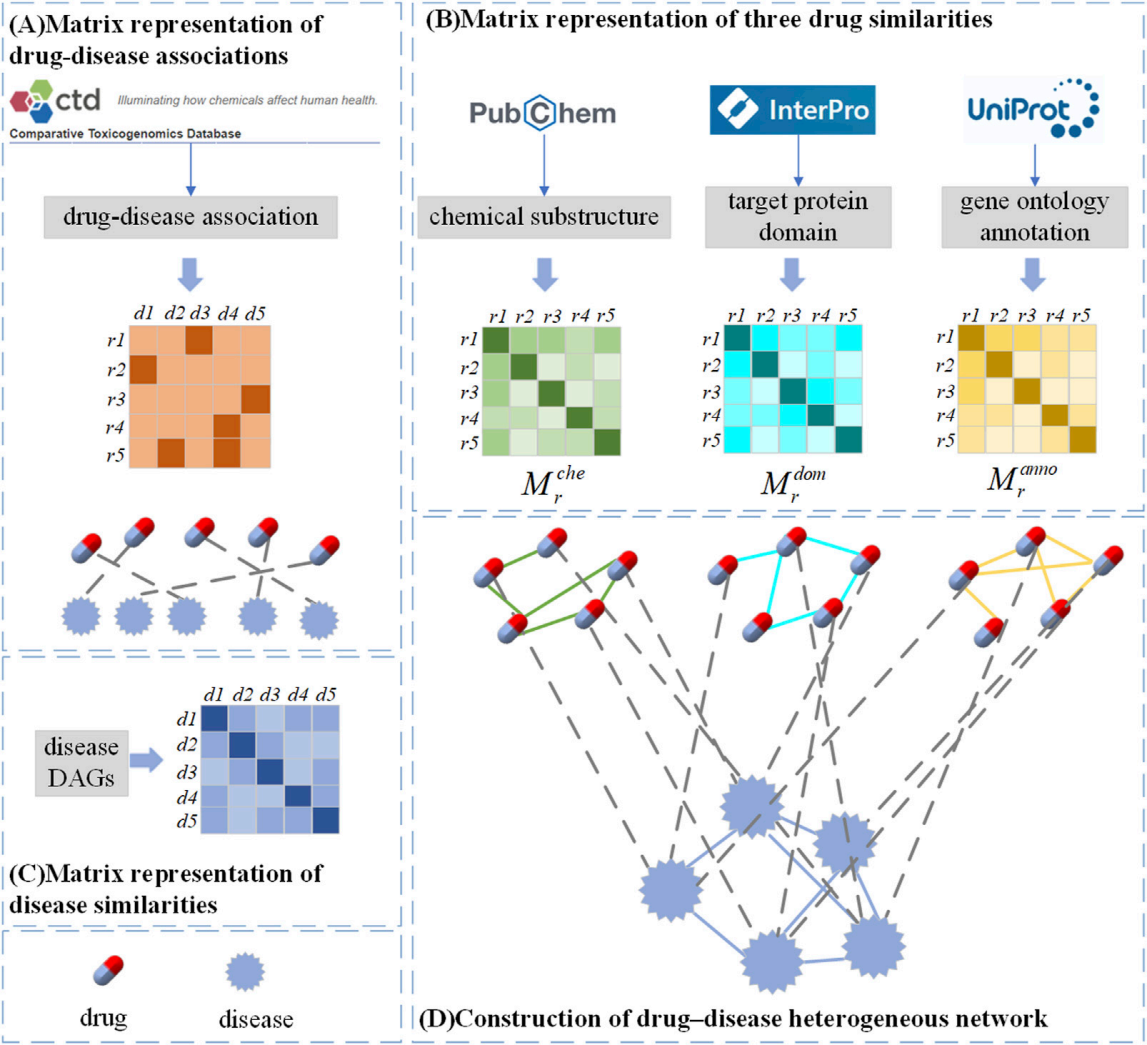
**(A) **matrix representation of drug-disease associations, **(B) **matrix representation of three drug similarities, **(C) **matrix representation of disease similarities, **(D) **construction of drug–disease heterogeneous network.

We build a drug-disease relationship matrix $M_{rd}\in R^{N_{r}\times N_{d}}$ (Figure1A) from the known drug-disease associations in the database, which records $N_{r}$ drugs and $N_{d}$ diseases connection situation. The rows of the matrix represent drugs and the columns represent diseases. If drug $r_{i}$ is associated with disease $d_{j}$ , then ${\left(M_{rd}\right)}_{i,j}=1$ , otherwise ${\left(M_{rd}\right)}_{i,j}=0$ .

#### 2.2.2 Drug Similarity Network

Considering the influence of biological knowledge of drugs on the prediction of drug-disease relationship, we introduce three different drug feature information, and multiple features can describe drug similarity from multiple different perspectives. Usually, the more chemical substructures two drugs have, the more similar their effects are. Similarly, when two drugs are present with more target proteins gene ontology annotation of domains or target proteins, which are often more similar (Ding et al., 2014). In previous studies (Xuan et al., 2019b), the Jaccard index and cosine similarity were commonly used to measure drug similarity. LAGCN (Yu et al., 2021) used these two methods separately to calculate drug similarity and found that the results of Jaccard index were slightly better than cosine similarity. Therefore, we use the Jaccard index to calculate the chemical substructure similarity of drugs, represented by a matrix $M_{r}^{che}$ . Similarly, the domain similarity and functional annotation similarity of drugs are represented by matrices $M_{r}^{dom}$ and $M_{r}^{anno}$ , respectively (Figure1B). In order to combine the information of different types of drug features, according to research (Liang et al., 2017), we project the three drug similarity matrices into a common latent subspace to get the final drug similarity matrix expressed as follows:

$$\begin{array}{c} M_{r}=\left\{\begin{array}{l} M_{r}^{che}={\left(M_{r}^{che}\right)}_{i,j}\\ M_{r}^{dom}={\left(M_{r}^{dom}\right)}_{i,j}\\ M_{r}^{anno}={\left(M_{r}^{anno}\right)}_{i.j} \end{array}\right.\in R^{N_{r}\times N_{r}} \end{array}$$ (1)

where ${\left(M_{r}\right)}_{i,j}$ represents the similarity value between drug $r_{i}$ and drug $r_{j}$ , and higher similarity values indicate higher functional similarity. $N_{r}$ represents the number of drugs.

#### 2.2.3 Disease similarity network

There are also similarities between diseases, and calculating disease similarity is crucial to building disease networks. According to previous studies (Wang et al., 2010), diseases can usually be represented by a directed acyclic graph (DAG), where nodes represent diseases and edges represent relationships between nodes. Each disease has associated disease terms in the DAG, and when two diseases have more of the same disease terms, they are often more similar. Here the graph of disease *d* is represented as $DAG_{d}=\left(d,V_{d},E_{d}\right)$ , where $V_{d}$ is the set of all ancestor nodes of *d*, including node *d* itself, and $E_{d}$ is the set of corresponding links. Define the contribution of disease *s* in $DAG_{d}$ to the semantics of disease *d* as follows:

$$\begin{array}{c} \left\{\begin{array}{l} D_{d}\left(d\right)=1\\ D_{d}\left(s\right)=\max\left\{\Delta *D_{d}\left(s^{\prime }\right)\left|s^{\prime }\in \begin{array}{ccc} children & of & \left.s\right\}\begin{array}{cc} if & s\neq d \end{array} \end{array}\right.\right. \end{array}\right. \end{array}$$ (2)

where Δ is the semantic contribution factor of the edge connecting disease *d* and its sub-disease *d'*, which ranges from 0 to 1, and is usually set to 0.5. Then the semantic value of disease *d* is defined as $DV\left(d\right)=\sum_{s\in V_{d}}D_{d}\left(s\right)$ .The semantic similarity of the two diseases was measured by considering their relative positions in the MeSH database (http://www.ncbi.nlm.nih.gov/) DAG. This disease similarity was also used in our study and represented by matrix $M_{d}\in R^{N_{d}\times N_{d}}$ (Figure1C). Then the semantic similarity of diseases is defined as follows:

$$\begin{array}{c} {\left(M_{d}\right)}_{i,j}=\frac{\sum s\in V_{d_{i}}\cap V_{d_{j}}\left(D_{d_{i}}\left(s\right)+D_{d_{j}}\left(s\right)\right)}{DV\left(d_{i}\right)+DV\left(d_{j}\right)} \end{array}$$ (3)

where ${\left(M_{d}\right)}_{i,j}$ represents the similarity value between disease $d_{i}$ and disease $d_{j}$ , and $N_{d}$ represents the number of diseases.

To enable our model to learn more and deeper drug-disease-related information, we construct heterogeneous networks through drug-disease similarity networks and drug-disease association networks. The three drug similarity networks reflect the similarity between two drugs from different perspectives, so we construct a drug-disease heterogeneous network based on the similarity of three drugs (Figure 1D). Each network contains two types of nodes (drug nodes, disease nodes) and three types of edges (drug-drug, disease-disease, and drug-disease). The nodes in the drug similarity network and the disease similarity network are connected, but there is no edge between the two networks. We add corresponding edges between these two networks based on known drug-disease associations. Specifically, if ${\left(M_{rd}\right)}_{i,j}=1$ , then add an edge between drug $r_{i}$ and disease $d_{j}$ . The adjacency matrix of the constructed heterogeneous network is expressed as follows:

$$\begin{array}{c} M_{h}=\left[\begin{array}{cc} M_{r} & M_{rd}\\ {M_{rd}}^{T} & M_{d} \end{array}\right]\in R^{\left(N_{r}+N_{d}\right)\times \left(N_{r}+N_{d}\right)} \end{array}$$ (4)

where ${M_{rd}}^{T}$ is the transpose of $M_{rd}$ .

### 2.3 Method

**FIGURE 2:**
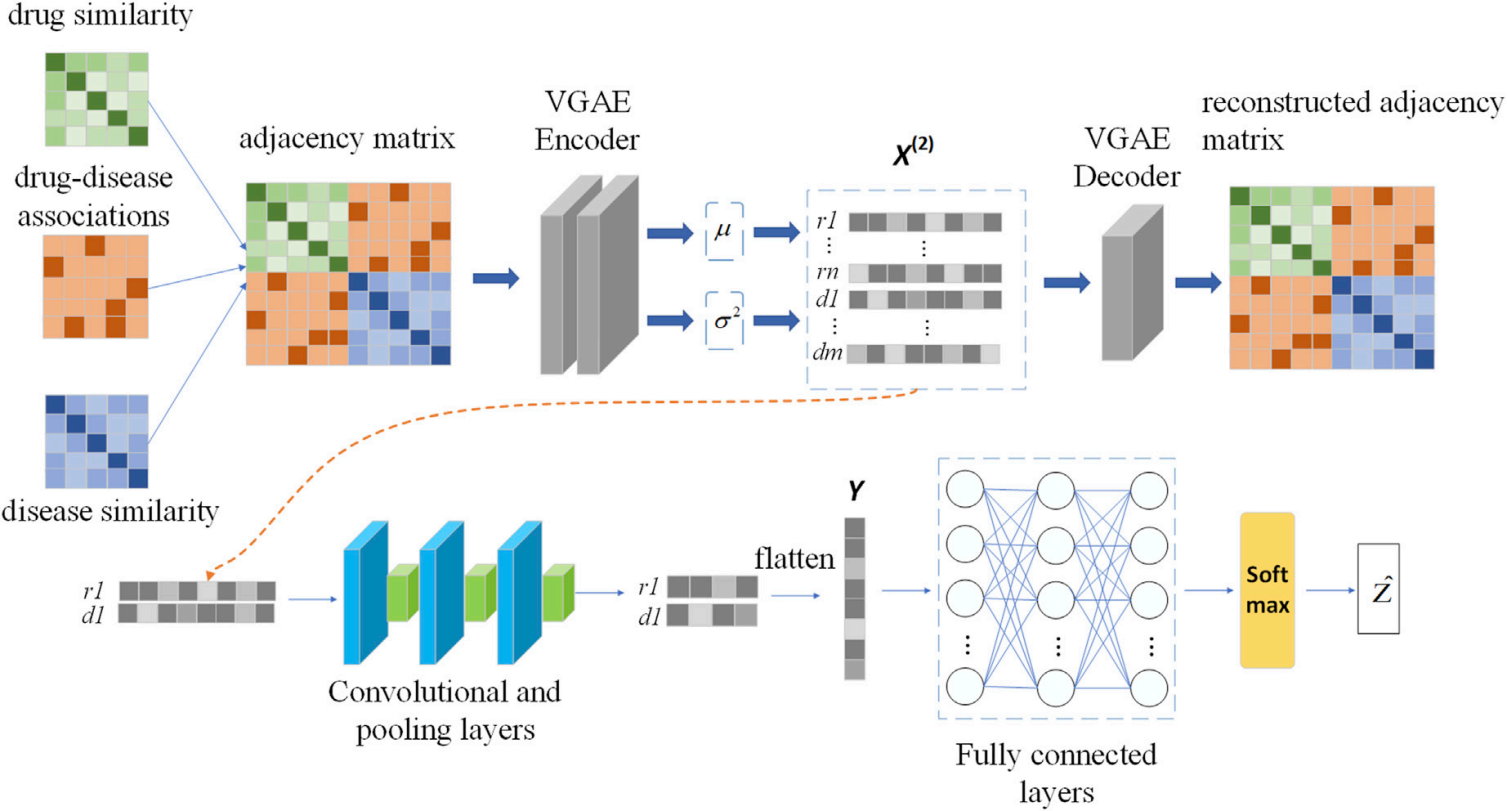
The workflow of VGAEDR model.

In this section we build a drug-disease relationship prediction model VGAEDR based on Variational Graph Autoencoders (VGAE) and CNN. The input of the model is a drug-disease heterogeneous network, which learns the network information of drug-disease through a graph variational autoencoder ensemble and generates latent low-dimensional feature matrix. The feature matrix is then fed into a multi-layer convolutional module to obtain the final drug-disease feature representation. Finally, the association probability of the drug-disease pair is obtained through the fully connected layer and the softmax layer. The structure of the VGAEDR model is shown in Figure 2.

#### 2.3.1 Graph representation learning based on VGAE

Variational Graph Autoencoder (VGAE) is an unsupervised learning framework for graph data structures based on Variational Autoencoder (VAE). The model contains two networks, an inference network and a generative network, which can also be interpreted as an encoder and a decoder. The encoding layer uses graph convolution to encode the known graph to learn a distribution of node vector representations, sample the node vector representations from the distribution, and then reconstruct the graph using an inner-product decoder.

In order to learn the network information formed by multiple connections between drug and disease nodes, we take the adjacency matrix $M_{h}$ and feature matrix *X* of the drug-disease heterogeneous network as the input of VGAE. The VGAE encoder part is a two-layer graph convolutional network (GCN), the first GCN layer learns the low-dimensional feature vectors of nodes from the network, and the second GCN layer generates the distribution of node feature representations. $M_{h}$ only contains the neighbor information of the drug or disease node, while ignoring the information of the node itself. Therefore, we add its own connection to each drug and disease node, let $A=M_{h}+I$ , I is the identity matrix, $I\in R^{\left(\left(N_{r}+N_{d}\right)\times \left(N_{r}+N_{d}\right)\right)}$ . Next, define the initial feature matrix for the drug and disease nodes as:

$$\begin{array}{c} X^{\left(0\right)}=\left[\begin{array}{cc} 0 & M_{rd}\\ M_{rd}^{T} & 0 \end{array}\right]\in R^{\left(N_{r}+N_{d}\right)\times \left(N_{r}+N_{d}\right)} \end{array}$$ (5)

Then, the low-dimensional feature representation of drug disease nodes can be obtained through the first GCN layer:

$$\begin{array}{c} X^{\left(1\right)}=ReLU\left(D^{-\frac{1}{2}}AD^{-\frac{1}{2}}{X^{\left(0\right)}W}_{0}\right) \end{array}$$ (6)

where $W_{0}\in R^{\left(N_{r}+N_{d}\right)\times d_{0}}$ is the weight matrix of the first GCN layer, and *d*_*0*_ is the dimension of the embedding. The second GCN layer learns the mean μ and variance σ represented by the low-dimensional vector corresponding to each node through the mean-variance calculation module:

$$\begin{array}{c} \mu =ReLU\left(D^{-\frac{1}{2}}AD^{-\frac{1}{2}}X^{\left(1\right)}W_{1}\right) \end{array}$$ (7)

$$\begin{array}{c} \log\sigma =ReLU\left(D^{-\frac{1}{2}}AD^{-\frac{1}{2}}X^{\left(1\right)}W_{2}\right) \end{array}$$ (8)

where μ and σ share the weight matrix $W_{0}$ of the first GCN layer, $W_{1}\in R^{\left(N_{r}+N_{d}\right)\times d_{1}}$ and $W_{2}\in R^{\left(N_{r}+N_{d}\right)\times d_{2}}$ are the weight matrices of μ and σ , respectively, *d*_*1*_ and *d*_*2*_ are their corresponding embedding dimensions. Then, the feature matrix representation $X^{\left(2\right)}\in R^{\left(N_{r}+N_{d}\right)\times \left(d_{1}+d2\right)}$ is obtained by sampling in $N\left(\mu ,\sigma^{2}\right)$ . $X^{\left(2\right)}$ can be divided into upper and lower parts, namely the drug node feature part (upper half) and the disease node feature part (lower half), as shown in Figure 2. $X_{i}^{\left(2\right)}$ is the *i*th row of the matrix $X^{\left(2\right)}$ , representing the feature vector of the *i*th node. Then the feature vectors of the drug node *r*_*i*_ and the disease node *d*_*i*_ are expressed as follows:

$$\begin{array}{c} r_{i}=\left\{X_{i}^{\left(2\right)}|i\in \left[1,n\right]\right\} \end{array}$$ (9)

$$\begin{array}{c} d_{i}=\left\{X_{i}^{\left(2\right)}|i\in \left[n+1,m\right]\right\} \end{array}$$ (10)

where $n=N^{r}$ , $m=N^{r}+N^{d}$ .

The decoder reconstructs the adjacency matrix by computing the inner product between the latent variables generated by the encoder:

$$\begin{array}{c} \hat{A}=\sigma \left({X^{\left(2\right)}}^{T}X^{\left(2\right)}\right) \end{array}$$ (11)

The decoder is defined as follows:

$$\begin{array}{c} p\left(A_{ij}=1\left|X_{i}^{\left(2\right)},X_{j}^{\left(2\right)}\right.\right)=\sigma \left(X_{i}^{{\left(2\right)}^{T}}X_{j}^{\left(2\right)}\right) \end{array}$$ (12)

where $\sigma \left(\cdot \right)$ is the sigmoid activation function and *T* represents the transpose.

Optimization: In order to minimize the difference between the generated graph and the original graph, the loss function of the VGAE module includes the distance metric between the generated graph and the original graph, and the nodes represent the divergence of the vector distribution and the normal distribution. Furthermore, we optimize the loss function of VGAE with the Adam function. The loss function is defined as follows:

$$\begin{array}{c} L^{vgae}=E_{q\left(X^{\left(2\right)}\left|X,A\right.\right)}\left[\log p\left(A\left|X^{\left(2\right)}\right.\right)\right]-KL\left[q\left(X^{\left(2\right)}\left|X,A\right.\right)\|p\left(X^{\left(2\right)}\right)\right] \end{array}$$ (13)

where $E_{q\left(X^{\left(2\right)}\left|X,A\right.\right)}\left[\log p\left(A\left|X^{\left(2\right)}\right.\right)\right]$ is the cross-entropy function, $KL\left[q\left(\cdot \right)\|p\left(\cdot \right)\right]$ is the KL divergence between $p\left(\cdot \right)$ and $q\left(\cdot \right)$ .

#### 2.3.2 CNN-based feature dimension reduction

We apply VGAE to a drug-disease heterogeneous network to learn feature representations for drug and disease nodes. Next, a convolutional neural network (CNN) is used to mine deeper feature representations of drug-disease nodes. The feature representation learning of drug node r_1_ and disease node d_1_ is shown in Figure 2.

The convolution module contains three convolution layers and pooling layers, and the number of filters increases layer by layer. The number of filters in the second layer of convolution is twice that of the first layer, and the number of filters in the third layer of convolution is three times that of the first layer. The length and width of the filters are *l* and *w*, respectively, and the number of filters in the first layer of convolution is *n*_*conv*_. We pad zeros around the drug-disease node feature matrix $X^{\left(2\right)}$ to learn the boundary information of $X^{\left(2\right)}$ , which is then used as the input of the convolution module. The filter $F_{conv}\in R^{l\times w\times n_{conv}}$ scans $X^{\left(2\right)}$ to obtain a set of feature maps *M*. We denote the area when we move the filter from the upper left corner of $X^{\left(2\right)}$ to the *i*th row and *j*th column as $X_{conv}^{\left(2\right)}\left(i,j\right)$ , then *M*_*k*_ is the feature map of $X^{\left(2\right)}$ obtained after the *k*th filter scan. $X_{conv}^{\left(2\right)}\left(i,j\right)$ and *M*_*k*_ are defined as follows:

$$\begin{array}{c} X_{conv}^{\left(2\right)}\left(i,j\right)=X^{\left(2\right)}\left(i:i+l,j:j+w\right)\in R^{l\times w} \end{array}$$ (14)

$$\begin{array}{c} M_{k}\left(i,j\right)=\sigma \left(W_{k}*X_{conv}^{\left(2\right)}\left(i,j\right)+b_{k}\right) \end{array}$$ (15)

where $W_{k}$ and $b_{k}$ are the weight matrix and bias vector of the *k*th filter, respectively, and σ is the nonlinear activation function Relu. To extract more important features and alleviate overfitting, we apply max pooling to *M*_*k*_. In the pooling layer, the length and width of the window are *p*_*l*_ and *p*_*w*_, respectively. The pooling result is *M*_*pool*,__*k*_, then the elements of its *i*th row and *j*th column are defined as follows:

$$M_{pool,k}\left(i,j\right)=\max\left(M_{k}\left(i:i+p_{l},j:j+p_{w}\right)\right)$$ (16)

Similarly, *M*_*pool, k*_ gets the latent representation *U* of drug and disease nodes after going through the second and third convolutional layers and max pooling layers, and then flattens it into a vector *Y*. *Y* takes as input to a fully connected layer, which is similar to a traditional neural network, where all neurons are connected to each other and the output is the result of the weighted sum of all outputs given by previously connected neurons. We used 1024 nodes in the first two fully connected layers, each followed by a dropout layer with rate 0.1. The third layer consists of 512 nodes. Finally, a softmax layer is applied to Y to obtain the association probability Ẑ of the drug-disease pair.

$$\begin{array}{c} \hat{Z}=soft\max\left(WY+b\right) \end{array}$$ (17)

where *W* and *b* are the weight matrix and bias vector, respectively. As a binary classification task, we use a cross-entropy loss function to evaluate the error between the true association and the predicted outcome. The loss function is as follows:

$$\begin{array}{c} L^{cnn}=-\left[Z\log\hat{Z}+\left(1-Z\right)\log\left(1-\hat{Z}\right)\right] \end{array}$$ (18)

where *Z* is the true label value.

## 3 Experiments

### 3.1 Experiment settings and evaluation metrics

We use 5-fold cross-validation to evaluate the predictive performance of our model and other models. All known drug-disease associations were considered positive samples and randomly divided into five equal parts. Since the number of negative samples in our dataset was significantly more than the number of positive samples and they all randomly sampled negative samples when compared with other methods, in order to unify the standard, we randomly selected some unobserved drug-disease associations equal to the number of positive samples as negative samples and randomly divided them into five equal parts. Positive samples and negative samples are taken together as a sample set. Next, we take four samples from the positive samples and negative samples respectively as the cross-validation set, and the remaining one sample in each of the two sample sets is used as the independent test set, thus ensuring that there is no overlap between the cross-validation set and the independent test set. We use a cross-validation set for model pre-training and parameter analysis in our experiments, and an independent test set for performance comparison with other baseline methods. 5 times of training and testing are performed, and the test results of these 5 times are averaged.

We mainly use seven evaluation metrics: area under the receiver operating characteristic (ROC) curve (AUC), area under the precision-recall curve (AUPR), F1_SCORE, accuracy, specificity, precision, and recall. The AUC value can reflect the probability that the positive samples predicted by the model are ahead of the negative samples, and when the distribution of positive and negative samples changes, its value can remain basically unchanged. Therefore, this evaluation index can reduce the interference caused by different test sets. A more objective measure of the performance of the model itself (Ling et al., 2003). The two indicators of precision and recall are usually used to evaluate the analysis effect of the binary classification model. F1_SCORE is defined as the harmonic mean of precision and recall. Our model predicts the association probability for each drug-disease pair in an independent test set, and if the association probability is above a given threshold, the sample is predicted to be a positive sample, otherwise it is a negative sample. The ROC curves are drawn based on TPR and FPR at different thresholds, and the true positive rate (TPR) and false positive rate (FPR) at the corresponding thresholds are as follows:

$$\begin{array}{c} TPR=\frac{TP}{TP+FN} \end{array}$$ (19)

$$\begin{array}{c} FPR=\frac{FP}{TN+FP} \end{array}$$ (20)

where *TP(TN)* is the number of samples correctly identified as positive samples (negative samples) and *FP(FN)* is the number of false positive samples (negative samples).

The number of positive samples in our dataset is much smaller than the number of negative samples, and there is a problem of imbalanced data categories. However, AUC is often less informative than AUPR when evaluating some data imbalance problems (Saito and Rehmsmeier, 2015). Therefore, we also use AUPR as an important evaluation metric, and the PR curve is drawn based on precision and recall. Precision and recall are defined as follows:

$$\begin{array}{c} Precision=\frac{TP}{TP+FP} \end{array}$$ (21)

$$\begin{array}{c} Recall=\frac{TP}{TP+FN} \end{array}$$ (22)

### 3.2 Parameter sensitivity analysis

**FIGURE 3:**
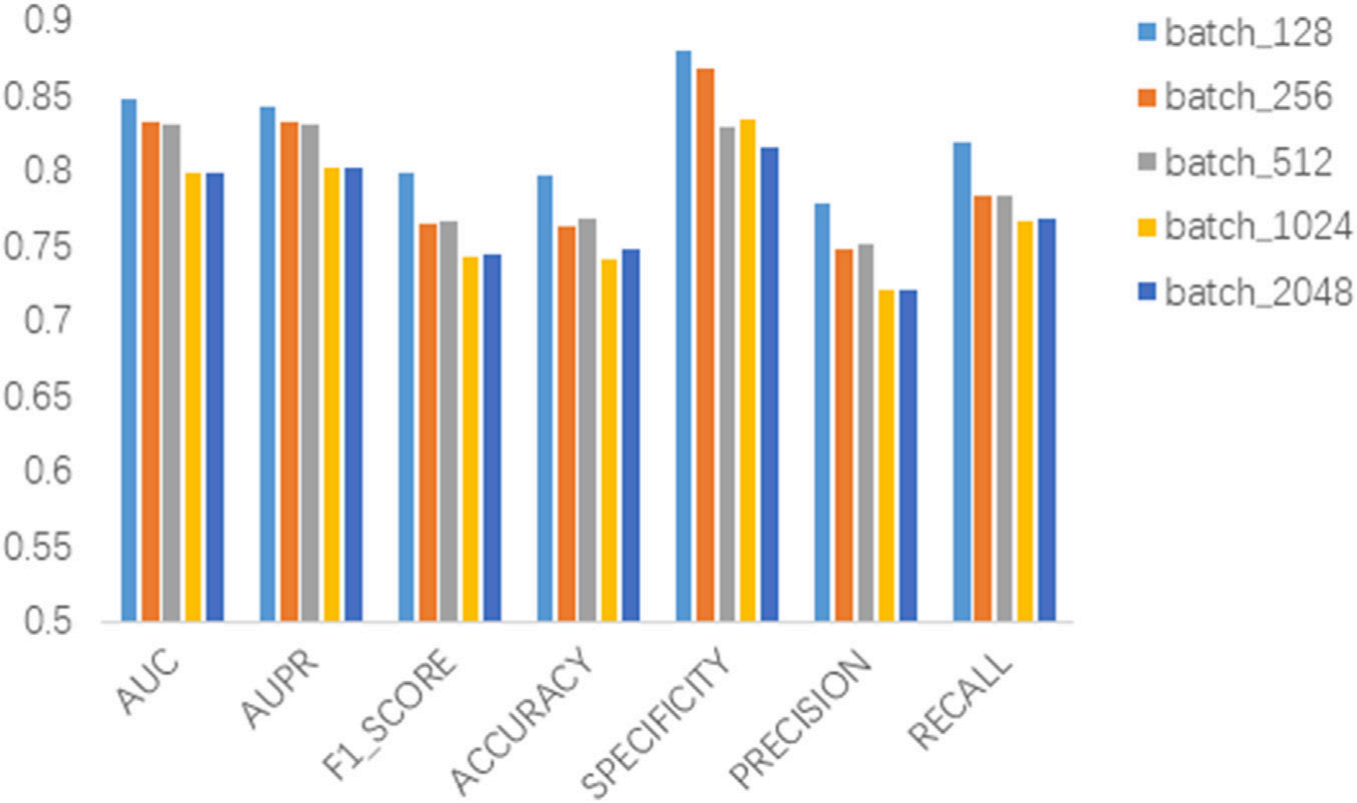
Effect of batch size.

**FIGURE 4:**
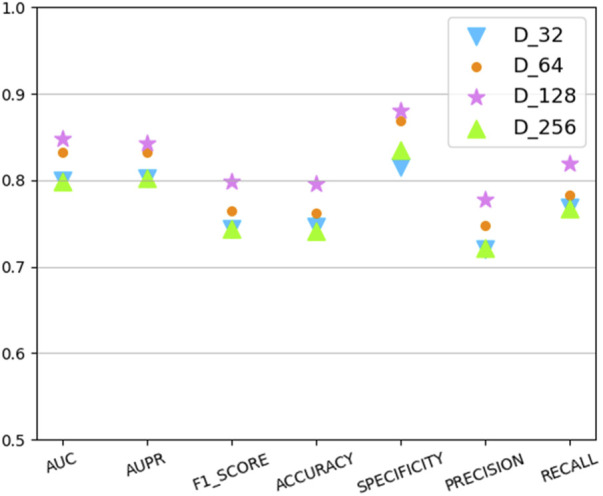
The effect of embedding dimension.

In this section we analyze the hyperparameter sensitivity of VGAEDR. Since VGAEDR is trained and tested in batches on the data, the choice of batch size may have different effects on the performance of the model. Under normal circumstances, if the batch size is too small, it will take a long time, and the gradient will oscillate seriously, which is not conducive to convergence; if the batch size is too large, the gradient direction of different batches will not change, and it is easy to fall into a local minimum. We tested the effect of different batch sizes on the model performance on the CTD dataset, and the experimental results are shown in Figure 3. The model achieves the best performance when the batch size is 128. It is worth noting that the embedding dimension *D* of the first GCN layer in the encoder of VGAE can contribute to the improvement of the model performance, we test on the CTD dataset, where *D* is (32, 64, 128, and 256), as shown in Figure 4, and finally we choose 128 as the best embedding dimension.

### 3.3 Ablation experiments

**FIGURE 5:**
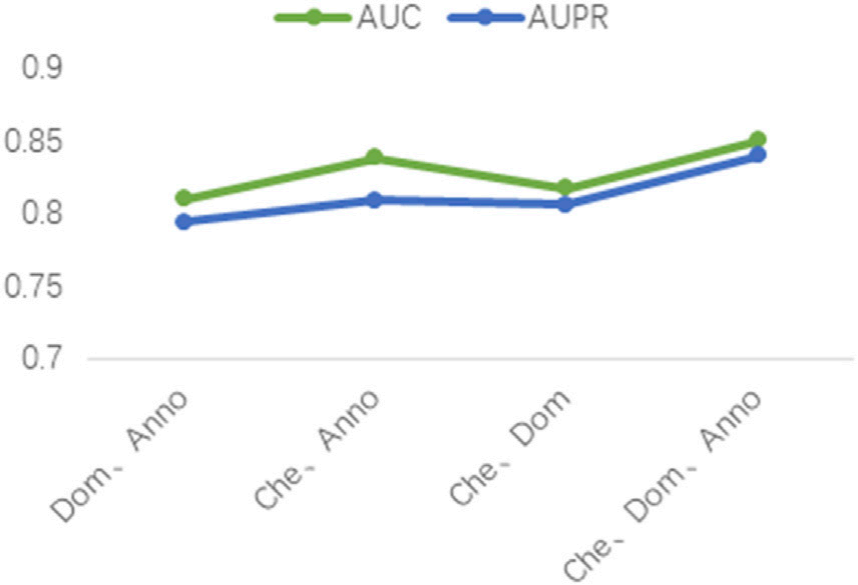
Results of the ablation studies.

Three different drug attribute data were used in our study, namely the chemical substructure of the drug, the domain of the drug target protein, and the gene annotation of the drug target protein. To validate the contribution of these three drug attribute data to our predictive model, we performed ablation experiments. The chemical substructure of the drug, the domain of the drug target protein, and the gene annotation data of the drug target protein are represented by Che, Dom, and Anno, respectively. As shown in Figure 5, firstly, the optimal results are achieved when training the model with Che, Dom, and Anno data simultaneously. Second, the AUC and AUPR of the model trained with Che and Dom were 0.0325 and 0.0341 lower than the model trained with all data. The models trained on Che and Anno have a drop of 0.0118 and 0.031 in AUC and AUPR, respectively, compared to the final model. Finally, the model achieved the lowest AUC and AUPR without Che. Obviously, the use of medicinal chemical substructure data has the greatest impact on model training, and the use of drug target protein domains and gene annotations has similar effects on model performance. A possible reason for this is that drugs generally have more defined chemical substructures, and they have fewer experimentally confirmed targets (Xuan et al., 2021). Ablation experiments show that training the model with data related to drug attributes is helpful for the predictive performance of the model.

### 3.4 Comparison of graph representation methods

**FIGURE 6:**
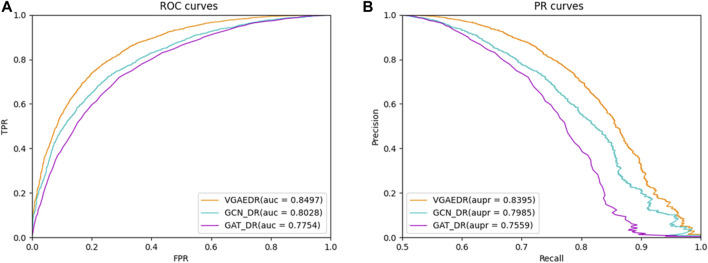
**(A)** The ROC curves of different graph representation learning, **(B)** The PR curves of different graph representation learning.

Besides VGAE, there are other graph representation learning methods that can also learn network representations of biomolecules in bioinformatics networks, such as GCN (Kipf and Welling, 2016) and GAT (Velikovi, 2018). To investigate their performance differences with VGAE, we integrate them with the convolutional neural network part of the VGAEDR model to obtain two variant models GCN_DR and GAT_DR. The three models are trained and tested on Dataset1, and the experimental results are shown in Figure 6. VGAEDR achieves the state-of-the-art performance, which indicates that VGAE is more suitable for learning network representations of drug-diseases. GCN_DR may be due to the fact that GCN is too smooth and the performance is mediocre. The poor performance of GAT_DR may be caused by the fact that GAT does not fully utilize the edge information in the drug-disease network.

### 3.5 Comparison with other methods

To validate the performance of the VGAEDR model, we compare with five state-of-the-art drug-disease association prediction methods on three datasets, such as DeepDR (Zeng et al., 2019), SCMFDD (Zhang et al., 2018), LRSSL (Liang et al., 2017), BNNR (Yang et al., 2019) and GRGMF (Zhang et al., 2020). These methods are mainly divided into two categories: methods based on heterogeneous networks and methods based on matrix factorization. To make the comparison results more convincing, we train and test all methods on the same dataset, while each comparison method uses the best parameter settings from the corresponding literature. Below we briefly describe the five comparison methods:

1) DeepDR Zeng et al. (2019) integrates drug-disease associations and multiple drug similarity networks into a heterogeneous network to predict novel drug-disease associations through multimodal deep autoencoders and collective variational autoencoders.
2) SCMFDD Zhang et al. (2018) projects drug-disease associations into two low-rank spaces, revealing latent features of drugs and diseases, and then introduces drug-feature-based similarity and disease semantic similarity as constraints on drugs and diseases in the low-rank space.
3) LRSSL Liang et al. (2017) fuses medicinal chemical information, drug target domain information and target annotation information to predict novel drug-disease associations based on Laplacian regularized sparse subspace learning.
4) BNNR Yang et al. (2019) integrates drug-drug, drug-disease, and disease-disease networks into a drug-disease heterogeneous network, and then uses the bounded kernel norm regularization (BNNR) method to complete the drug-disease under low-rank hypothesis matrix.
5) GRGMF Zhang et al. (2020) formulated a generalized matrix factorization model that considers the neighborhood information of each node when learning the latent representation of each node, and can learn the neighborhood information of each node adaptively.

As shown in Table 2, VGAEDR achieves the best performance on all metrics in the CTD dataset compared with the other five methods. To verify the robustness of VGAEDR, we also conduct experiments on Dataset1 and Dataset2. Table 3 shows the performance comparison results of VGAEDR and the other five methods on Dataset1. The AUC value of VGAEDR is the highest of 0.8497, which is 1.93% higher than the second-ranked DeepDR and 3.36% higher than the third-ranked SCMFDD. DeepDR can only integrate drug-related feature information due to the structure of collective variational autoencoder model. However, without disease feature information, the prediction performance of the model is often affected. SCMFDD only uses a single drug feature information to build the prediction model, when we have multiple drug features, we can calculate the similarity of different drug features. Combining different information generally improves performance, which may be the reason why VGAEDR performs better than SCMFDD. BNNR also only considers a single drug (disease) similarity, and performs matrix completion on heterogeneous networks, which leads to poor performance of the model on datasets with large amounts of data to a certain extent. LRSSL has an obvious drawback that the regularization of disease similarity may fail when the number of diseases is too small, thus affecting the prediction performance. GRGMF ranks relatively low in performance, which may be due to the fact that it does not mine deeper representations of the drug-disease network and the fact that matrix factorization models usually perform moderately well when dealing with sparse matrices. In other evaluation indicators, VGAEDR also achieved the best results. The improved performance of VGAEDR is mainly attributed to its deep learning capabilities, as well as its ability to comprehensively learn and mine drug-disease heterogeneous networks. Table 4 shows the performance comparison results of VGAEDR and the other five methods on Dataset2. Except recall, VGAEDR outperforms these five comparison methods in all other evaluation metrics. However, compared with the results on Dataset1, the AUC of VGAEDR is reduced by 0.91%, the AUPR is reduced by 0.93%, the F1_SCORE is reduced by 1.2%, and other indicators are also reduced. We reasoned that this might be because the known drug-disease associations on Dataset2 were far less than those on Dataset1.

**TABLE 2:** Performance of comparison methods on CTD.

	AUC	AUPR	F1_SCORE	Accuracy	Specificity	Precision	Recall
VGAEDR	0.8484	0.8423	0.7982	0.7962	0.8805	0.7777	0.8198
DeepDR	0.8286	0.8243	0.7533	0.7542	0.839	0.7262	0.7825
SCMFDD	0.8173	0.8105	0.7412	0.7458	0.8051	0.6916	0.7984
LRSSL	0.8186	0.8225	0.7701	0.7693	0.8146	0.7763	0.764
BNNR	0.8154	0.8067	0.7361	0.7336	0.8133	0.6905	0.7881
GRGMF	0.8037	0.8018	0.7526	0.7531	0.7966	0.7335	0.7727

**TABLE 3:** Performance of comparison methods on Dataset1.

	AUC	AUPR	F1_SCORE	Accuracy	Specificity	Precision	Recall
VGAEDR	0.8497	0.8395	0.7963	0.8013	0.8789	0.7764	0.8172
DeepDR	0.8304	0.8289	0.7521	0.756	0.8452	0.7178	0.7898
SCMFDD	0.8161	0.809	0.7394	0.7331	0.8006	0.6979	0.7862
LRSSL	0.8132	0.8261	0.7652	0.7619	0.8274	0.7394	0.7929
BNNR	0.8139	0.8042	0.7348	0.7308	0.7859	0.7009	0.775
GRGMF	0.8042	0.8029	0.7532	0.753	0.7939	0.7186	0.7913

**TABLE 4:** Performance of comparison method on Dataset2.

	AUC	AUPR	F1_SCORE	Accuracy	Specificity	Precision	Recall
VGAEDR	0.8406	0.8302	0.7843	0.7812	0.856	0.7733	0.7956
DeepDR	0.8143	0.8156	0.7436	0.7457	0.8318	0.6921	0.8173
SCMFDD	0.8065	0.8074	0.7255	0.723	0.7993	0.6655	0.7974
LRSSL	0.8053	0.817	0.7475	0.7486	0.813	0.7079	0.7918
BNNR	0.8129	0.8049	0.7312	0.7378	0.7762	0.6878	0.7805
GRGMF	0.7938	0.7835	0.7468	0.7495	0.8004	0.7108	0.7866

**FIGURE 7:**
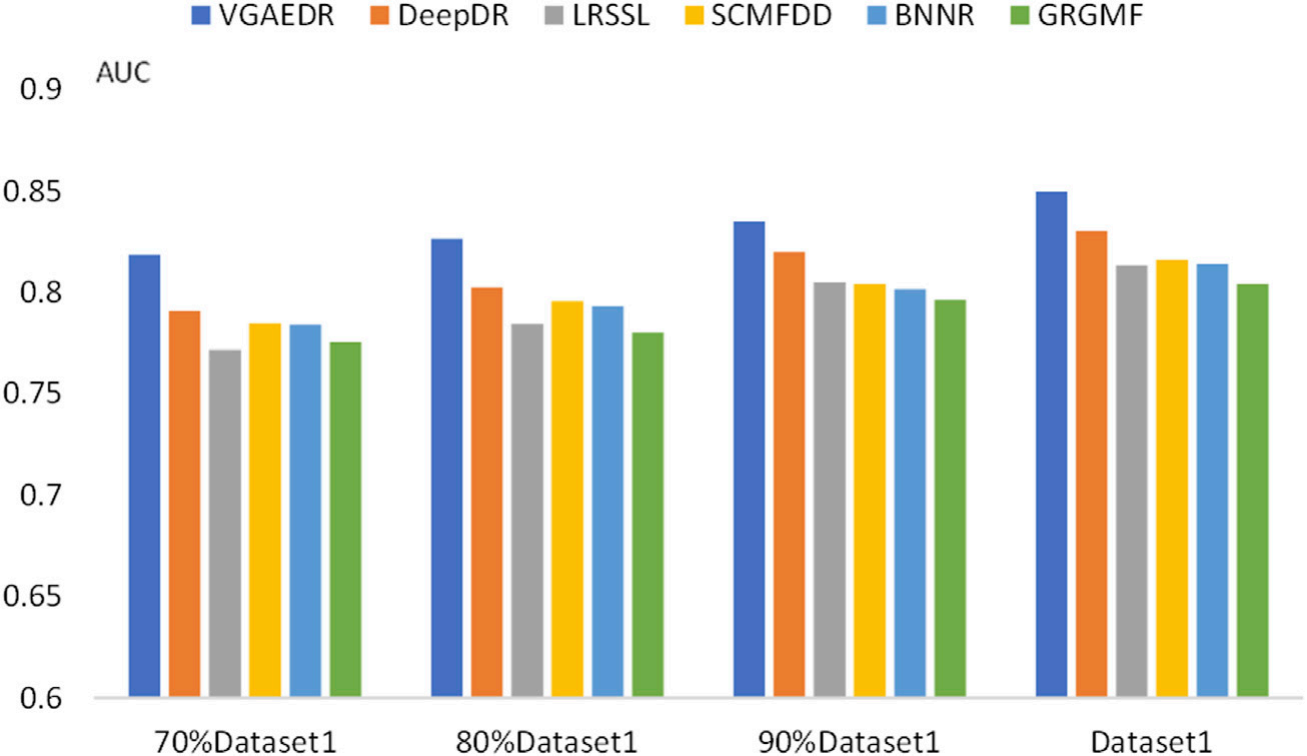
AUC of methods based on different fractions of known associations.

**FIGURE 8:**
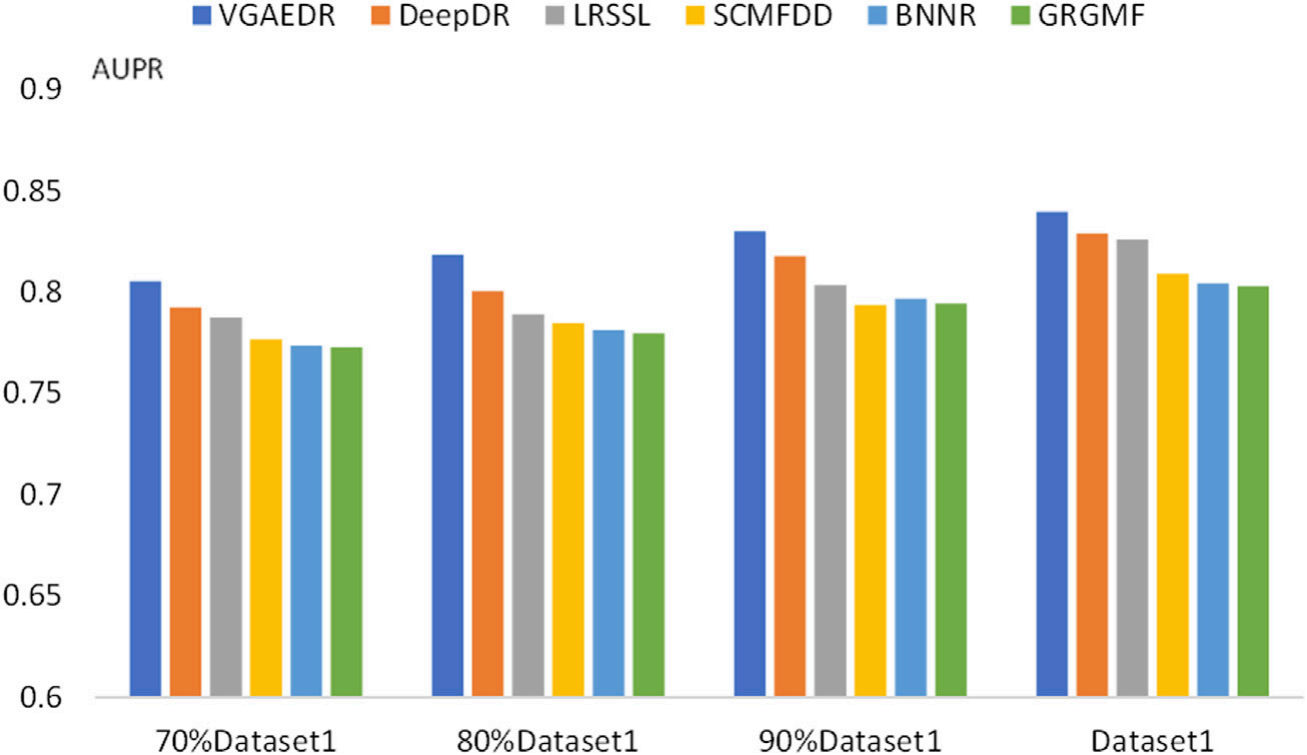
AUPR of methods based on different fractions of known associations.

To validate our inferences, specifically, that the number of known drug-disease associations is an important factor in predicting potential drug-disease associations, which may significantly affect the performance of the method. We took Dataset1 as a sample, and randomly selected 70%, 80%, and 90% of them, which is equivalent to obtaining four datasets with different numbers. We compare the performance of VGAEDR and five other methods on these four datasets, as shown in Figures 7, 8, as the number of known drug-disease associations increases, the AUC and AUPR of all methods basically becomes higher, where VGAEDR achieves the best performance. This suggests that more drug-disease associations lead to better predictive performance of the model.

### 3.6 Case studies

Coronavirus disease 2019 (COVID-19), which has spread globally and has a significant impact on the global economy and health, is caused by severe acute respiratory syndrome coronavirus 2 (SARS-CoV-2). Although the medical diagnosis of COVID-19 is rapid and effective, no effective treatment currently exists. Therefore, potential therapeutic drugs should be screened. Drug repositioning is considered a strategy that can speed up the treatment process. We predicted the top 10 possible anti-covid-19 drugs, as shown in Table 5, and 6 of them can be found in the relevant literature. Since the three datasets we used do not contain data on COVID-19 and antiviral drugs, we used the HDVD database mentioned in Zhang et al. (2022). According to Li et al. (2021), glycyrrhizic acid (GA) is clinically an anti-inflammatory drug against inflammatory stress caused by pneumonia, and the combination of glycyrrhizic acid and vitamin C can serve as a potential treatment for COVID-19 Treatment options. Zhao et al. (2021) mentioned in their study that GA has antiviral effects on different viruses, including SARS-related coronaviruses. According to its characteristics, GA is considered as a promising novel drug candidate against SARS-CoV-2 by testing alone or in combination with other drugs. Favipiravir is an established treatment for influenza and is being more explored for its role in treating COVID-19. It is the first oral antiviral drug approved for mild to moderate COVID-19. Studies that have been done in China, Japan, and Russia suggest that favipiravir is a promising treatment for this disease (Joshi et al., 2021). In a study Manabe et al. (2021), favipiravir induced viral clearance within 7 days and contributed to clinical improvement within 14 days. The results suggest that favipiravir has a high potential to treat COVID-19, especially in patients with mild to moderate disease. Vaccination is also a way to protect against viruses, and Burnett (Burnett et al., 2017) studied the global impact of rotavirus vaccination on childhood hospitalization and diarrheal mortality. Arouche et al. (2020) studied the molecular docking of Bcx4430 and five other potential pharmacologically active inhibitor compounds that can be used clinically against the COVID-19 virus, Bcx4430 interacts with the main COVID-19 protease and the COVID-19 N3 protease inhibitor complex. In molecular docking studies, tenofovir was recently shown to bind to SARS-CoV-2 RNA polymerase (RdRp) with a binding energy comparable to that of natural nucleotides and to a similar extent to that of remdesivir. Therefore, tenofovir has recently been suggested as a potential treatment for COVID-19 (Harter et al., 2020). Umifenovir can prevent viral contact and penetration of host cells by avoiding fusion of viral lipid capsids to cell membranes, and can inhibit COVID-19 infection by interfering with SARS-COV-2 release from intracellular vesicles (Nojomi et al., 2020). Therefore, umifenovir is considered as one of the antiviral drugs that can effectively treat COVID-19 patients. Tacrolimus may be effective in the treatment of post-COVID-19 acute interstitial lung disease, but does not prevent the progression of pulmonary fibrosis (Ohgushi et al., 2022).

**TABLE 5:** Top 10 possible anti-COVID-19 drugs predicted by the VGAEDR.

Rank	Accession number	Drug name	2D structure	Evidence (PMID)
1	DB13751	Glycyrrhizic Acid	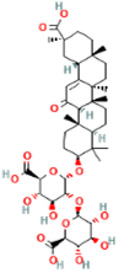	32662814, Li et al. (2021) 33930273, Zhao et al. (2021)
2	DB12466	Favipiravir	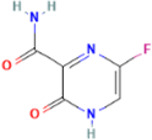	33130203, Joshi et al. (2021) 34044777, Manabe et al. (2021)
3	DB11676	Bcx4430	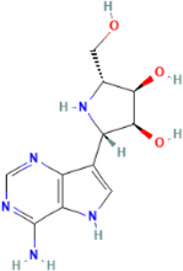	32711596, Arouche et al. (2020)
4	DB01015	Sulfamethoxazole	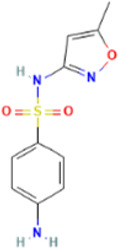	NA
5	DB05102	Rupintrivir	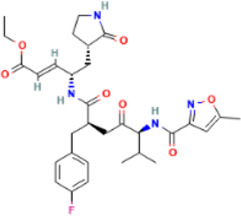	NA
6	DB00300	Tenofovir Disoproxil	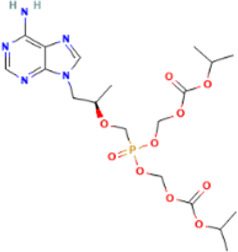	32394344, Harter et al. (2021)
7	DB13609	Umifenovir	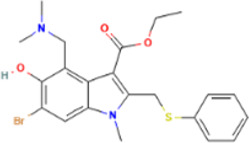	33317461, Nojomi et al. (2020)
8	DB00290	Bleomycin	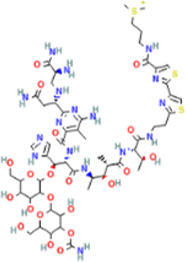	NA
9	DB00864	Tacrolimus	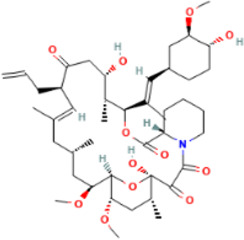	34866097, Ohgushi et al. (2022)
10	DB01029	Irbesartan	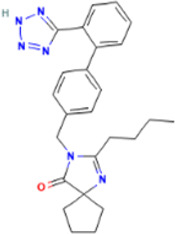	NA

Therefore, the case study demonstrates that VGAEDR can identify novel drug-disease associations and effectively predict drugs that may fight COVID-19.

## 4 Conclusion

In this article, we propose a drug repositioning method, VGAEDR, based on variational graph autoencoders and heterogeneous networks. First, a drug-disease heterogeneous network is constructed based on three different drug feature similarities, disease semantic similarities, and known drug-disease associations. Then, a Variational Graph Autoencoder (VGAE) module for learning heterogeneous networks is established. The heterogeneous network is used as the input of the VGAE module, and then it learns its latent low-dimensional feature representation and generates the reconstructed network. Finally, a multi-layer convolutional neural network module is built to further learn its low-dimensional feature representation. We input the feature representations finally learned by the two modules into fully connected layers and softmax layers to predict drug-disease associations. Ablation experiments show that using multiple drug feature data can improve the predictive performance of the model. The comparison results with other five methods on the three datasets demonstrate the excellent performance of our model. VGAEDR also achieves the best results in datasets containing different numbers of drug-disease associations, while demonstrating that the greater the number of known drug-disease associations, the better the predictive performance of the model. We conducted case studies on existing drug and disease data, and predicted the top 10 possible anti-COVID-19 drugs, six of which were verified by other literatures. It was demonstrated that VGAEDR is a reliable drug repositioning method.

In the future work, since we only use the disease semantic similarity as the disease feature in this paper, we will consider more disease similarity information in the subsequent work, such as disease phenotype similarity and disease Gaussian kernel similarity, to integrate this disease feature information for better drug repositioning. We are also ready to collect and collate more drug-disease association data from more databases and literature to train the model and thus improve its predictive ability. In addition, we only have reliable positive samples (known drug-disease associations), negative samples are selected by random sampling, and more algorithms for selecting negative samples will be considered in future work. At present, we can only obtain information from relevant literature and reports to verify new drug-disease associations. In the future, if there is an opportunity, we hope to cooperate with researchers in the field of biochemistry to verify the candidate drugs for a disease we predict through a series of wet experimental methods.

## Data Availability

The original contributions presented in the study are included in the article/Supplementary Material, further inquiries can be directed to the corresponding author.
